# Characterization of Temperature-Dependent Kinetics of Oculocutaneous Albinism-Causing Mutants of Tyrosinase

**DOI:** 10.3390/ijms22157771

**Published:** 2021-07-21

**Authors:** Samuel A. Wachamo, Milan H. Patel, Paul K. Varghese, Monika B. Dolinska, Yuri V. Sergeev

**Affiliations:** National Eye Institute, National Institutes of Health, Bethesda, MD 20892, USA; sw6bj@virginia.edu (S.A.W.); milanh.patel@hmhn.org (M.H.P.); paulv@vt.edu (P.K.V.); dolinskam@nei.nih.gov (M.B.D.)

**Keywords:** human recombinant tyrosinases, OCA1B mutations, temperature-dependent kinetics, Van’t Hoff analysis, L-DOPA binding, decreased dopachrome in mutant variants

## Abstract

Human tyrosinase (Tyr) is a glycoenzyme that catalyzes the first and rate-limiting step in melanin production, and its gene (*TYR*) is mutated in many cases of oculocutaneous albinism type 1 (OCA1). The mechanisms by which individual mutations contribute to the diverse pigmentation phenotype in patients with OCA1 have only began to be examined and remain to be delineated. Here, we analyze the temperature-dependent kinetics of wild-type Tyr (WT) and two OCA1B mutant variants (R422Q and P406L) using Michaelis–Menten and Van’t Hoff analyses. Recombinant truncated human Tyr proteins (residues 19–469) were produced in the whole insect *Trichoplusia Ni* larvae. Proteins were purified by a combination of affinity and size-exclusion chromatography. The temperature dependence of diphenol oxidase protein activities and kinetic parameters were measured by dopachrome absorption. Using the same experimental conditions, computational simulations were performed to assess the temperature-dependent association of L-DOPA and Tyr. Our results revealed, for the first time, that the association of L-DOPA with R422Q and P406L followed by dopachrome formation is a complex reaction supported by enthalpy and entropy forces. We show that the WT has a higher turnover number as compared with both R422Q and P406L. Elucidating the kinetics and thermodynamics of mutant variants of Tyr in OCA1B helps to understand the mechanisms by which they lower Tyr catalytic activity and to discover novel therapies for patients.

## 1. Introduction

Mutations in the tyrosinase gene (*TYR*) cause oculocutaneous albinism type 1 (OCA1), an autosomal recessive disorder characterized by a lack of melanin biosynthesis or reduced melanin pigment in the hair, skin, and eyes. OCA1, the most common type of albinism, is caused by bi-allelic mutations in the *TYR* gene with an estimated prevalence of 1:40,000 globally [1]. Depending on the severity of the mutation, OCA1 is further divided into two subtypes, OCA1A and OCA1B. OCA1A is the most severe type of OCA1, leading to a complete lack of melanin production due to loss of tyrosinase (Tyr) activity. OCA1A patients have a lifelong absence of pigmentation and present white hair and eyelashes, pale skin, and translucent irides [2]. Unlike OCA1A, OCA1B is marked by reduced Tyr activity and melanin production as compared with unaffected people. OCA1B patients have hypopigmentation in the skin and hair with the ability to tan, accumulating melanin pigment with age. However, both subtypes of OCA1 have common features, including nystagmus, foveal hypoplasia with reduced visual acuity, and abnormal decussation of ganglion cell axons at the optic chiasm [1]. Despite the identification of more than 350 mutations in the *TYR* gene, currently, there is no effective treatment for OCA1, making it an area of active investigation [1,2,3]. A recent study by Teramae et al. that used HeLa cells to express mutant Tyr demonstrated that chemical chaperone therapy offers a promising treatment modality for recovering Tyr activities of OCA1A patients with certain types of missense mutations of *TYR* [4].

However, the thermodynamics and kinetics of mutant variants of Tyr have yet to be elucidated to understand the mechanisms by which mutations contribute to the diverse pigmentation phenotype in OCA1 and to recover Tyr activity in patients. Previous in vivo [5] and in vitro [6] studies have shown that there are temperature-sensitive mutant variants of Tyr that cause OCA1. For instance, R422Q leads to a temperature-sensitive trafficking defect restraining the translocation of the mutant Tyr into melanosomes, where melanogenesis takes place. At 37 °C, R422Q is retained in the endoplasmic reticulum (ER) and degraded by proteasomes, resulting in no pigmentation, whereas at 31 °C it is successfully translocated into the melanosome, producing pigment. This phenomenon is observed in patients with OCA1B, presenting white hair in the warmer areas (scalp and axilla) and progressively darker hair in the cooler areas (extremities) [5,7,8]. The role of temperature in Tyr structure and function needs further exploration to better understand mechanisms involved in OCA1. Moreover, to the best of our knowledge, the role of temperatures in the production of dopachrome, especially in mutant variants, has not been reported.

Human Tyr is one of the three key enzymes involved in the melanogenesis pathway, which results in the production of melanin pigments. Tyr is a copper-containing glycoenzyme that catalyzes the initial and rate-limiting step in the cascade of reactions leading to melanin production from tyrosine [9]. This metalloenzyme is made of 529 amino acids, including an N-terminal signal peptide sequence, cysteine-rich subdomain (the EGF domain), C-terminal transmembrane alpha-helix domain, two copper-binding sites, and seven sites of N-glycosylation. The homology model of Tyr is shown in the Supplementary Materials Figure S11. Post-translational glycosylation ensures proper Tyr maturation, stability, and function. The active site of Tyr contains two copper atoms bridged by an aquo (hydroxo) ligand and ligated to six histidine residues (H180, H202, and H211 at CuA and H363, H367, and H390 at CuB) [3,9,10]. The stability of the copper ions within the active site of Tyr is reliant upon the presence of a bridging dioxygen molecule. As shown in previous computational experiments, the removal of this bridge may lead to active site disarray with a lack of coordination from the six histidine residues [11]. Tyr catalyzes the first two steps of the melanogenesis pathway, hydroxylation of L-tyrosine to L-3, 4-dihyroxyphenyl-alanine/L-DOPA (monophenolase activity/rate-limiting step), and the subsequent oxidation of L-DOPA to L-dopaquinone (diphenolase activity). Then, L-dopaquinone is spontaneously converted to an orange-brown intermediate known as dopachrome through intramolecular cyclization and redox exchange [12,13]. Tyr diphenol oxidase activity can be monitored by measuring the dopachrome production at 475 nm [14]. Tyr also catalyzes the oxidation of downstream intermediate 5,6-dihydroxyindole (DHI) to indole-5,6-quinone and 5,6-dihydroxyindole-2-carboxylic acid (DHICA) to indole-5,6-quinone carboxylic acid [15]. As an enzyme that catalyzes the rate-limiting step of the melanogenesis pathway, Tyr plays a central role in the production of eumelanin and pheomelanin.

Our group previously purified and characterized recombinant truncated human wild type tyrosinase (WT) and mutant variants from T. ni. larval biomass [1,2,16]. These characterizations revealed that there is a link between Tyr conformational stability and its enzymatic activity. We have shown that the WT is a soluble monomeric glycoprotein, which is post-translationally modified in the ER by the addition of several N-linked glycans. Glycosylation plays a vital role in Tyr maturation, stability, and translocation from the ER to the cytoplasm. Deglycosylated mutants of Tyr exhibit a moderate to dramatic decrease in specific L-DOPA activity, depending on the degree of deglycosylation [1,2]. We have also shown that OCA1A related mutants (T373K and R77Q) and OCA1B related mutants (R402Q, R422W, R422Q, and P406L) influence protein folding, stability, and intrinsic activity. The K_m_ values of WT and OCA1B mutant variants were similar, implying similar L-DOPA binding affinities, but the enzyme turnover was decreased for mutant variants [1]. Subsequently, lower turnover led to decreased dopachrome production and melanin. However, our previous assessments of the diphenol oxidase reaction of mutant variants of Tyr were only done at 31 and 37 °C. Furthermore, we have demonstrated a correlation between changes in protein stability and specific activity by using urea-induced equilibrium unfolding/refolding of WT and mutant variants. The refolded WT, R402Q, and R422Q showed native-like response curves, while R422W and P406L exhibited hysteresis. WT and R422W proteins displayed similar free energy changes (ΔG), whereas R402Q, R422Q, and P406L exhibited lower stability (higher ΔG). Thus, OCA1B-causing mutations inhibit the folding pathway of Tyr to the lowest energy conformation, which in turn could decrease catalytic activity due to perturbed conformation [1].

Recently, we examined the temperature-dependent kinetics of the WT using Michaelis–Menten kinetics and Van’t Hoff analysis, demonstrating that the association of L-DOPA with the WT is a spontaneous enthalpy-driven reaction [17]. In the present study, we characterize the temperature-dependent kinetics and thermodynamic signatures of WT and two OCA1B mutants of Tyr, R422Q and P406L, using diphenol oxidase activities at 28, 31, 37, and 43 °C. Under the same experimental conditions, we performed computational simulations of the association of L-DOPA and Tyr. To obtain the kinetic and thermodynamic parameters, we used Michaelis–Menten and Van’t Hoff analyses. We also assessed the role of temperature in the production of dopachrome through the diphenol oxidase reaction. Our results revealed, for the first time, that the association of L-DOPA with R422Q and P406L is a complex reaction supported by enthalpy and entropy forces. We further showed that the WT had a higher turnover number as compared with both R422Q and P406L. Moreover, the production of dopachrome increased with increasing temperature for WT, R422Q, and P406L, but it was significantly higher for the WT at all temperatures examined. Elucidating the kinetics and thermodynamics of mutant variants of Tyr in OCA1B helps to understand the mechanisms by which they lower Tyr catalytic activity. This, in turn, could accelerate the search for novel compounds that can recover Tyr activity in OCA1B patients. In the future, in addition to in vitro studies, in vivo studies of similar nature coupled with investigation of other mutants would advance our understanding of mechanisms in OCA1B and lead to discoveries of novel therapies for OCA1B patients.

## 2. Results

### 2.1. Protein Purification

The WT, R422Q, and P406L proteins were purified as previously described [10,16]. Briefly, immobilized metal affinity chromatography (IMAC) was followed by two steps of size exclusion chromatography (SEC). Tyr colorimetric assay with L-DOPA was performed to identify fractions containing the proteins of interest. The purification steps were monitored by SDS-PAGE and Western blot to assess purity and identity, respectively. In the Supplementary Materials, Figure S1 shows the SEC profile of Tyr after purification using a Superdex 200 Increase GL 10/300 (GE Healthcare, Silver Spring, MD, USA) or a Superdex 75 16/60 HR column (GE Healthcare, Silver Spring, MD, USA), SDS-PAGE, and Western Blot. The purification (Supplementary Materials Figure S1, Panel A and D) demonstrated that WT, R422Q, and P406L elute as monomeric proteins with estimated molecular weights of 58, 56, and 61 kDa, respectively. As shown in the Supplementary Materials Figure S1 (Panels B, C, E, and F), the SDS-PAGE illustrated a highly pure protein with a single band, while the Western Blot confirmed the identity of Tyr using anti-Tyr (T311) antibodies. In addition, the SDS-PAGE and Western Blot for the mutants revealed bands with molecular weights like that of the WT protein.

After each step of purification, fractions containing Tyr were identified by an assessment of the enzymatic activity of the analytes in the fractions, measuring the diphenol oxidase activity of Tyr [2]. The activity was measured spectrophotometrically at 475 nm using a 1:1 ratio of the analyte and 3 mM L-DOPA substrate. The production of an orange-brown dopachrome confirmed the presence of an active Tyr in a fraction.

### 2.2. Temperature-Dependent Kinetics

**Figure 1:**
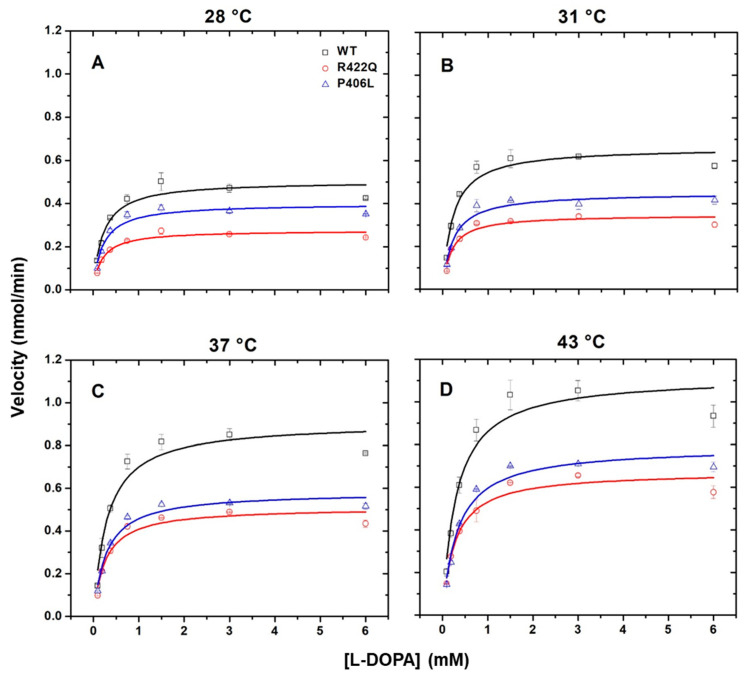
Kinetic analysis of WT and OCA1B-related mutants. Panels (**A**–**D**) show the Michaelis-Menten plots of diphenol oxidase activity of WT, R422Q, and P406L as a function of L-DOPA concentration measured at increasing temperatures. WT, R422Q, and P406L are shown in black, red, and purple, respectively. Each data point represents the average of duplicate measurements, with error bars representing standard deviations.

**Figure 2:**
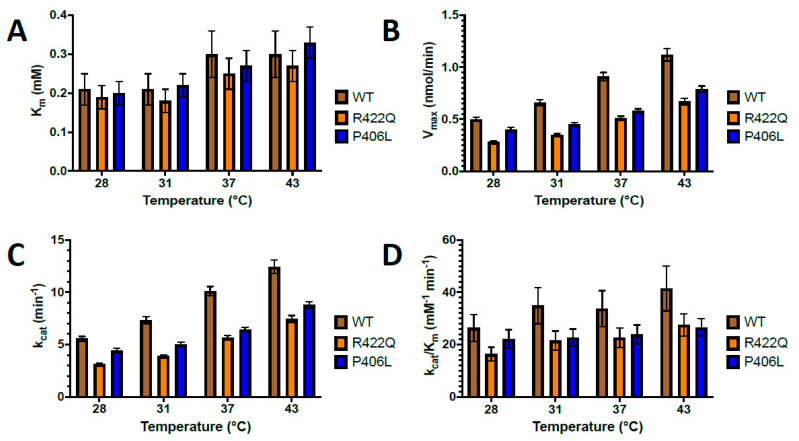
Kinetic parameters of WT and OCA1B-related mutants at increasing temperatures. The K_m_, V_max_, *k_cat_*, and enzyme efficiency increased with increasing temperature for both WT and mutant variants, as shown in panels (**A**–**D**), respectively. However, the WT exhibited higher V_max_, *k_cat_*, and enzyme efficiency as compared with mutant variants. WT, R422Q, and P406L are shown in brown, orange, and blue, respectively. Each data point represents the average of duplicate measurements, with error bars representing standard deviations.

Previous studies have shown that temperature regulates the production of melanin in the melanocytes and have confirmed the existence of temperature-sensitive mutants of tyrosinase [7,18,19]. However, the role of temperature in mutant variants of Tyr is not well understood. Here, we measured the temperature dependence of the catalytic activity of WT, R422Q, and P406L and examined their kinetics to understand the molecular mechanism of oculocutaneous albinism. The enzymatic activities were measured in vitro as described in the Methods section under Michaelis–Menten kinetics. The affinity constant K_m_ and maximal velocity V_max_ obtained from the Michaelis–Menten plots are shown in Figure 1, and then the additional kinetic parameters shown in Figure 2 and in the Supplementary Materials Table S1 were calculated.

The diphenol oxidase activity displayed increasing V_max_, *k_cat_*, and catalytic efficiency as temperature increased from 28 to 43 °C (Figure 2 and Supplementary Materials Table S1). The V_max_ (nmol/min) ranged from 0.50 ± 0.02 to 1.12 ± 0.06 for WT and showed decreased values from 0.28 ± 0.01 to 0.67 ± 0.03 for R422Q, and from 0.40 ± 0.02 to 0.79 ± 0.03 for P406L. The *k_cat_* (min^−1^) ranged from 5.55 ± 0.22 to 12.44 ± 0.66 for WT, from 3.11 ± 0.11 to 7.44 ± 0.33 for R422Q, and from 4.44 ± 0.22 to 8.78 ± 0.33 for P406L, suggesting that turnover number characterized by *k_cat_* is declined for mutant variants of Tyr. The catalytic efficiency (mM^−1^ min^−1^) ranged from 26.43 ± 5.14 to 41.47 ± 8.58 for WT and decayed for mutant variants from 16.37 ± 2.65 to 27.56 ± 4.26 for R422Q and from 22.20 ± 3.51 to 26.61 ± 3.38 for P406L. The V_max_, *k_cat_,* and efficiency values were higher for the WT as compared with the mutant variants at all temperatures, indicating a partial disruption of Tyr function caused by the mutations (Figure 2). In contrast, in both WT and mutant variants, the K_m_ values were similar for 28 and 31 °C but increased for 37 and 43 °C with similar magnitude (Figure 2 and Supplementary Materials Table S1). Unlike the other kinetic parameters, the K_m_ values were very similar for WT, R422Q, and P406L at each temperature examined.

### 2.3. The Role of Temperature in the Production of Dopachrome

**Figure 3:**
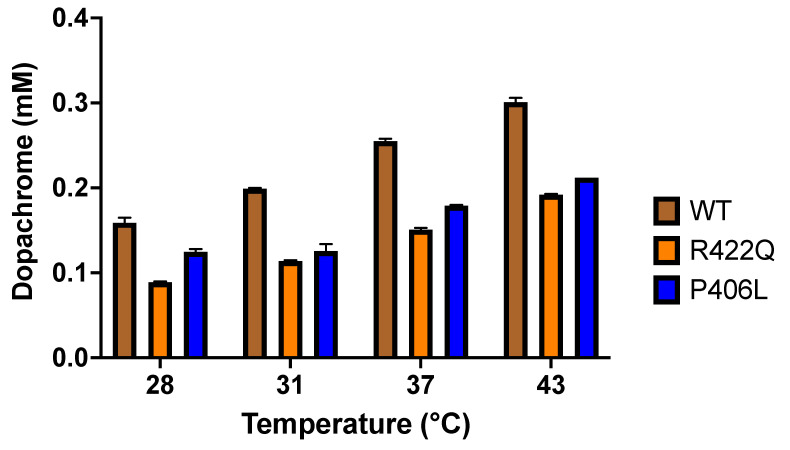
The role of temperature in the production of dopachrome. The diphenol oxidase activities were determined by using 0.05 mg/mL Tyr and 3 mM L-DOPA as a substrate. The absorbance measurements (mOD) of dopachrome production at 475 nm were obtained after running the reactions at the temperature conditions for 30 min. WT, R422Q, and P406L are shown in brown, orange, and blue, respectively. All experiments were performed in duplicates and error bars represent standard deviations.

We also assessed the role of temperature in the production of dopachrome through the diphenol oxidase reaction of Tyr. The diphenol oxidase activities of WT, R422Q, and P406L were measured at the temperature conditions described in the Methods section. The first 30 min of the diphenol oxidase reaction were used to analyze the production of dopachrome. These experiments revealed that the dopachrome increases with increasing temperature. Although the experiments were done for L-DOPA concentrations ranging from 0.094 to 6.0 mM, Figure 3 depicts the results of the diphenol oxidase activities for 3 mM L-DOPA substrate.

As shown in Figure 3 and in the Supplementary Materials Figure S2, the production of dopachrome is higher for the WT as compared with R422Q and P406L at all temperatures assessed here. However, in both the WT and mutant variants, the production of dopachrome is increasing linearly with temperature, according to the following equation D = P_d_ × T − D_o_ (Supplementary Materials Figure S2). Here D, P_d_, D_o_, and T are dopachrome production, dopachrome production rate, dopachrome production at 0 °C, and temperature (°C), respectively. Corresponding values of these parameters for WT protein and mutant variants are listed in Table 1.

In line with the kinetics data, the literature, and clinical presentation, we observed decreased dopachrome production in mutant variants as compared with the WT, signifying decreased activity of mutant Tyr. The decreased production of dopachrome, in turn, leads to reduced production of melanin.

### 2.4. Apparent Thermodynamic Signature of Dopachrome Formation

**Figure 4:**
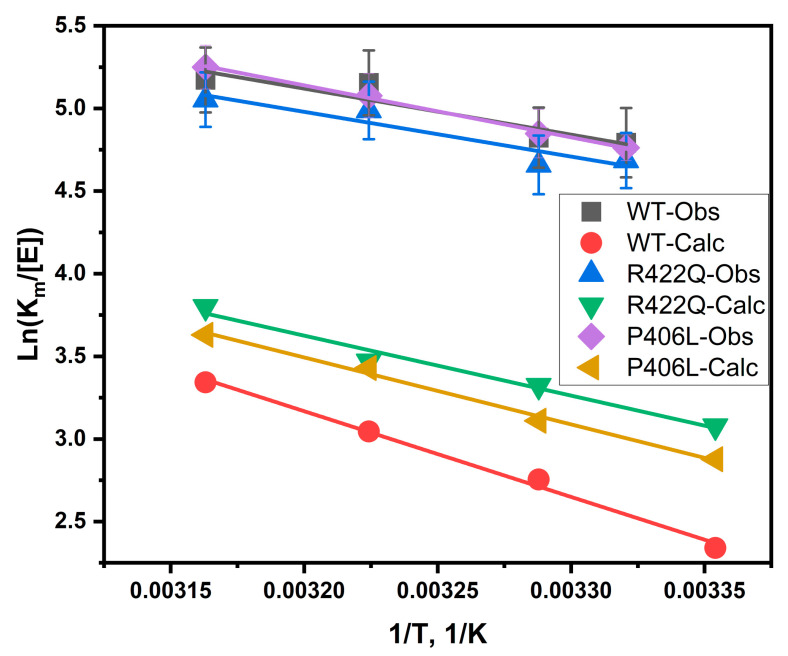
The Van’t Hoff plots of WT and mutant variants. Experimental (Obs) and calculated (Calc) data are shown for WT, R422Q, and P406L, respectively. These plots were derived from the fitting of the temperature-dependent kinetics data of the diphenol oxidase reactions and computational association data. All experiments were performed in duplicates and error bars represent standard deviations.

The apparent thermodynamic signature of dopachrome production was determined by the measurement of the Michaelis–Menten constant (K_m_) followed by an analysis using the Van’t Hoff equation. To explain the temperature properties of interaction, we assumed that L-DOPA associates with Tyr. The association indicates a negative trendline on the Van’t Hoff plot. Figure 4 shows Van’t Hoff plots slopes for WT, R422Q, and P406L diphenol oxidase reactions, which revealed that the reactions are exothermic (ΔH < 0). 

**Figure 5:**
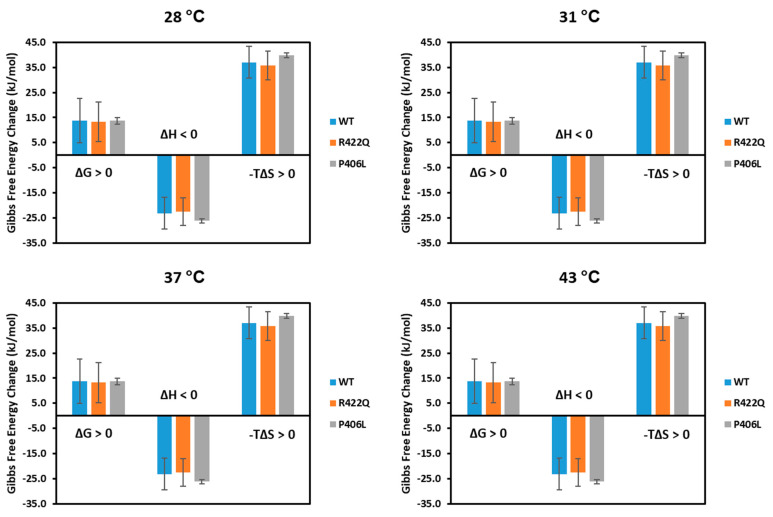
The apparent thermodynamic signatures of the diphenol oxidase reactions suggest reactions driven by enthalpy and entropy forces. WT, R422Q, and P406L are shown in blue, orange, and gray, respectively. All experiments were performed in duplicates and error bars represent standard deviations. The thermodynamic signatures for the docking simulations are shown in the Supplementary Materials Figure S3.

Figure 5 shows the apparent thermodynamic signatures of the diphenol oxidase reactions. 

Table 2 features the apparent ΔH and ΔS.

**Figure 6:**
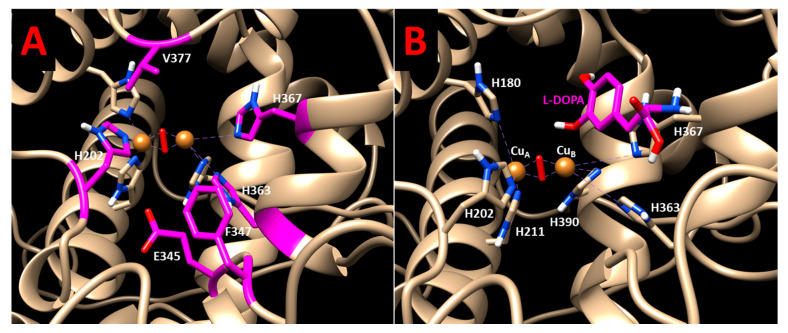
Molecular docking of L-DOPA and Tyr: (**A**) The residues of WT involved in substrate docking at all temperatures are shown in the active site. These residues include H202, E345, F347, H363, H367, and V377; (**B**) the L-DOPA molecule (magenta) is docked in the active site of WT. The computational docking shown here measures the binding energy of L-DOPA in the enzyme-substrate complex [Tyr*L-DOPA]. The two copper atoms at the active site of Tyr are shown by bronze spheres. They are coordinated by H180, H202, H211, H363, H367, and H390.

Computer simulations were conducted to assess the temperature-dependent association between Tyr and L-DOPA and to explain the experimental observations. Root mean square deviations graphs (RMSD) indicated that the simulations for WT, P406L, and R422Q were stable (Supplementary Materials Figure S10). L-DOPA was docked to Tyr at four different temperatures and docking results were only considered if the hydroxyl groups on the aromatic ring of L-DOPA were oriented towards the copper active site and if the YASARA output indicated that both copper atoms were contacting residues. Contacting residues were calculated within the dock_run.mcr script. The hydroxyl groups were situated within two water molecules (5.5 Å) of copper. Structural docking results are displayed in Figure 6 and demonstrate the binding of L-DOPA in the vicinity of the CuA and CuB atoms within the active site.

Four contacting receptor residues were found to be involved in ligand interactions for WT, R422Q, and P406L structures at all four temperatures assessed here: H202, E345, F347, and V377 (Supplementary Materials Figure S4). Graphs of the total number of hydrogen bonds in the protein structure for WT Tyr and R422Q are shown in the Supplementary Materials Figure S9. R422Q is expected to lose transient hydrogen bonds with surrounding residues including E409, E413, and E423. H363 and H367 were also involved for WT (Figure 6), and H367 and S375 for P406L. Dissociation constants (K_d_) were generated for each binding pose for Tyr (0.018–0.048 mM), R422Q (0.037–0.076 mM), and P406L (0.030–0.064 mM) (Supplementary Material Table S2). For each structure, K_d_ increases linearly with temperature, showing consistent results with experimental diphenol oxidase activity. The docking poses describe the Tyr*L-DOPA complex, and its temperature-dependent association produces negative trendlines on the Van’t Hoff plots of all three structures (Figure 4), revealing an exothermic reaction (∆H < 0). The Gibbs free energy was calculated for each temperature and, subsequently, the ΔΔG was determined for R422Q (1.498–1.066 kJ/mol for 28–43 °C), and P406L (0.914–0.614 kJ/mol for 28–43 °C). The results are displayed in Table 2 and in the Supplementary Materials Figure S3. Consistent with the experimental kinetics results, the association of Tyr and L-DOPA is a complex reaction supported by enthalpy and entropy forces.

## 3. Discussion

Temperature change is known to cause extracellular stress [19], but the role of temperature in melanin production, especially in OCA1B mutant variants of Tyr, has received little attention. Here, we characterize the temperature-dependent kinetics and thermodynamic signatures of Tyr and two OCA1B-related mutants, R422Q and P406L, at four temperatures by tracking the diphenol oxidase reaction. Utilizing the same experimental conditions, the activities and structures of WT and mutant variants were modeled using molecular dynamics and docking. The role of temperature in the production of dopachrome was also assessed through the diphenol oxidase reaction. For the first time, both experimental and computational results revealed that the association of L-DOPA with R422Q and P406L is a reaction supported by enthalpy and entropy forces. We further show that the WT has a higher turnover number as compared with both R422Q and P406L. Moreover, the production of dopachrome rose with increasing temperature for WT, R422Q, and P406L, but it was higher for the WT at all temperatures examined. Elucidating the kinetics and thermodynamics of OCA1B-related mutant variants helps us to understand the mechanisms by which they lower Tyr catalytic activity.

In our previous studies, we investigated the enzymatic activity of R422Q [1,2] (at 31 and 37 °C) and P406L [1] (at 37 °C). In line with our present study, we found that the *V_max_*, *k_cat_*, and enzyme efficiency values are lower for R422Q (*k_cat_* = 78.44 ± 9.69 s^−1^) and P406L (*k_cat_* = 72.17 ± 11.07 s^−1^) as compared with that of the WT (*k_cat_* = 128.59 s^−1^). The K_m_ values indicated that the binding affinity is slightly higher for R422Q (0.73 ± 0.12 mM) and P406L (0.65 ± 0.17 mM) as compared with that of the WT (0.85 ± 0.22 mM) [1]. This is also consistent with our present study but does not fit all data. For example, P406L has a slightly lower affinity at 31 and 43 °C as compared with that of the WT (Supplementary Materials Table S1). Overall, both previous and current studies show that R422Q and P406L are biochemically similar to the WT but display decreased enzymatic activity. In our present work, we determined the kinetic parameters of R422Q and P406L at more temperature points (28, 31, 37, and 43 °C) than done previously. We also examined the temperature-dependent production of dopachrome and the thermodynamic signatures of R422Q and P406L for the first time. In addition, we previously reported the temperature-dependent kinetics and thermodynamic signatures of WT [17]. Here, we investigated thermodynamic signatures of R422Q, P406L, and WT control using Van’t Hoff analysis (Figure 5). For the WT control, our results showed that the association of L-DOPA and Tyr is a favorable reaction, consistent with our previous publication as shown in Table 2 (ΔH < 0) [17]. The sign of ΔS is negative in our present study (WT ΔS = −0.12 ± 0.020) while it is positive in our recent paper (WT ΔS = 0.058 ± 0.020). We also noticed differences in the magnitudes of the thermodynamic parameters, but the overall trends were similar.

In the Michaelis–Menten kinetics experiment, K_m_ increased with temperature, implying decreased affinity of L-DOPA for Tyr at higher temperatures (Figure 1 and Supplementary Materials Table S1). This is consistent with the computational data which resulted in increasing K_d_ with temperature (Supplementary Materials Table S2). We assumed that the formation of product from the L-DOPA*Tyr complex occurs at a much slower rate as compared with the rate of dissociation of the complex. In this case, the K_m_ and K_d_ values are equal [17]. Since the K_m_ did not change significantly between WT and mutant variants at all temperatures assessed, the mutations likely do not affect the affinity of L-DOPA for Tyr at the active site. However, V_max_ and *k_cat_* decreased at all temperatures in mutant variants as compared with WT. The catalytic efficiency, which is the best value to represent Tyr’s overall ability to convert substrate to product, revealed that the mutant variants have lower efficiency (Figure 2 and Supplementary Materials Table S1). These results led us to infer that we could not exclude the possibility that the mutations might be affecting the activity of Tyr allosterically. Enzymes could have allosteric regulatory sites comprised of different residues away from an active site. These residues could be critical mediators of long-range communications and important contributors to the integrity of the enzyme structure [20]. Nonconservative replacement of these residues could result in a significant conformational shift and loss in catalytic efficiency. In addition, studies have shown that mutational perturbations consistently modulate the packing and dynamics of a significant fraction of protein residues, extending >10–15 Å from the mutated site. This long-range modulation of enzyme structure can affect protein stability and conformation, allowing allosteric modulation of function [21].

Allosteric instability affecting Tyr activity, in part, could be interpreted at the level of interatomic interactions. The P406L mutation allows the possibility of extending the alpha-helix (residues 384–403) containing H390, a copper coordinating histidine. Proline typically introduces a slight bend within alpha-helices due to the lack of a hydrogen bond (Supplementary Materials Figure S5). R422 forms three transient hydrogen bonds with E409, E413, and E423. The R422Q mutation removes the hydrogen bond accepting arginine from a glutamic acid pocket (Supplementary Materials Figure S6). To some extent, the change in Tyr activity could be attributed to the changes in structure and interactions due to R422Q and P406L mutations. These changes in mutants decrease V_max_ and the enzyme turnover. This phenomenon is shown in the Supplementary Materials Figures S7 and S8, where we investigated the kinetic parameters at five different time points over an hour of the diphenol oxidase reaction. Overall, the results from the temperature-dependent kinetics indicate that the mutant variants have decreased catalytic activity as compared with that of the WT.

The Michaelis–Menten kinetics of the diphenol oxidase reaction and in silico docking analysis of WT, R422Q, and P406L exhibit similar results with negative apparent enthalpy and positive entropy term as shown in Figure 5, Table 2, and the Supplementary Materials Figure S3. The balance between negative enthalpy (ΔH < 0) and positive entropy term (−TΔS > 0) drives the reaction to make the Gibbs free energy positive, therefore, the process is influenced by both enthalpy and entropy [22]. The negative entropy, which indicates the decrease of disorder in the system, could also be related to a decrease in the rotational and translational freedom of L-DOPA and dopachrome molecules, or a change in the conformational structure of dopachrome association due to different interactions. Although the overall enthalpy is negative and indicates a favorable diphenol oxidase reaction of WT and mutant variants, ionic and hydrophobic interactions might contribute to the enthalpy and entropy changes [23]. In summary, both methods, Michaelis–Menten kinetics and in silico docking, show that the association of L-DOPA and Tyr is a (ΔG > 0) reaction supported by enthalpy and entropy forces.

The change in the Tyr activity and the effect of the thermodynamic shift between WT and mutant variants are demonstrated by the significant change in dopachrome production through the diphenol oxidase reaction (Figure 3 and Table 1). The WT and mutant variants both showed increased production of dopachrome with increasing temperature. As shown in the Supplementary Materials Figure S2, the production of dopachrome increased linearly with temperature, according to the following equation D = P_d_ × T − D_o_. However, R422Q and P406L displayed lower dopachrome production at all temperatures assessed here. Dopachrome is produced by the interaction of L-DOPA, the substrate of the diphenol oxidase reaction, with Tyr, the enzyme that catalyzes the reaction to release dopaquinone from the enzyme-substrate complex. This is followed by the formation of dopachrome from dopaquinone through spontaneous intramolecular cyclization and redox exchange. Subsequent reactions eventually convert the dopachrome to melanin [12,13]. In the Michaelis–Menten kinetics, we measured spectrophotometrically the production of dopachrome at 475 nm, while our docking simulations delineated the association of L-DOPA and Tyr at the first stage of the diphenol oxidase reaction. Nevertheless, both the kinetics and in silico docking analyses demonstrated similar results, negative enthalpy and entropy (Table 2). As we have shown in our recent study [17], this implies that in silico docking, a successful model of the first step of the diphenol oxidase reaction correlates with dopachrome changes measured at 475 nm at the last step of the reaction.

**Figure 7:**
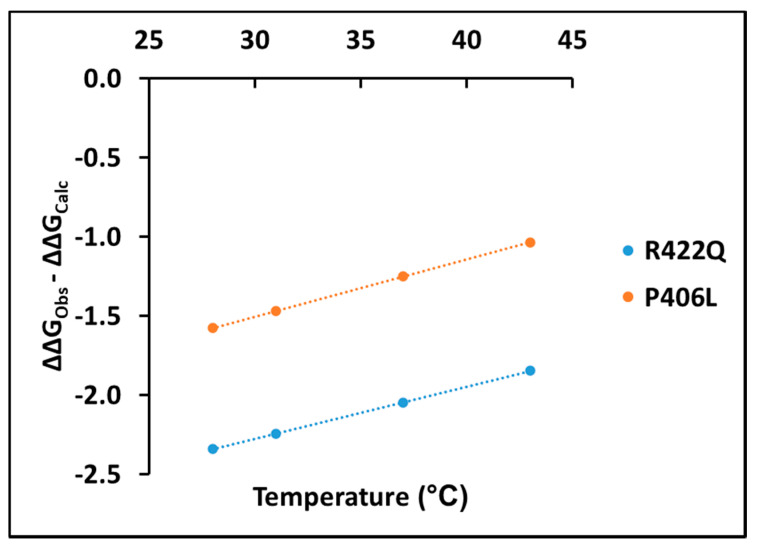
The difference between experimental ΔΔG_Obs_ and computational ΔΔG_Calc_ as a function of temperature (°C). Here, ΔΔG represents the free energy change obtained by subtracting the ΔG of the WT from the ΔG of mutant variants at different temperatures. The blue (E = 0.033T − 3.265, R^2^ = 1.000) and orange (E = 0.036T − 2.583, R^2^ = 1.000) lines are for R422Q and P406L, respectively.

Experimentally, measuring the association of L-DOPA with Tyr and determining the thermodynamic parameters behind this association is complicated because of the transient steps involved in the diphenol oxidase reaction. Here, we suggest taking into consideration the change in ΔΔG values between experimental and computational data to get a more accurate understanding of the dopachrome association in diphenol oxidase reaction. The changes in ΔΔG values between experimental and computational data are shown for R422Q and P406L in the Supplementary Materials Table S3. These values show that the change in ΔΔG is negative, suggesting stabilization by dopachrome. In both R422Q and P406L, the change in ΔΔG values between experimental and computational data increases linearly with temperature (adjusted R^2^ = 1.00) according to the following equation E = E_d_ × T − E_o_ (Figure 7).

Here, *E*, *E_d_*, *E_o_*, and *T* denote the change in ΔΔG (ΔΔG_Obs_ – ΔΔG_Calc_), rate of change in ΔΔG, change in ΔΔG at 0 °C, and temperature (28, 31, 37, or 43 °C), respectively. Corresponding values of these parameters for R422Q and P406L are listed in the Supplementary Materials Table S4. The positive trend line shown in Figure 7 implies a decrease in dopachrome-like product stability as the temperature increases. Furthermore, the higher values of free energy change for P406L in Figure 7 demonstrate that P406L is more unstable than R422Q. Therefore, the change in ΔΔG values could provide an easy method for elucidating the energetics of dopachrome associations, measuring dopachrome production spectrophotometrically, and assessing the association of L-DOPA and Tyr using docking simulations.

The analysis of the link between protein stability and ligand binding could be important for understanding the mechanisms involved in a decreased catalytic activity of mutant variants. This also might suggest potentially new treatments for the recovery of tyrosinase catalytic activity in mutant variants. Indeed, a recent study by Teramae et al. using HeLa cells to express mutant Tyr demonstrated that chemical chaperone therapy offered a promising treatment modality for recovering Tyr activity of OCA1A patients with certain types of missense mutations of *TYR* [4]. The same approach could potentially be used in OCA1B to restore tyrosinase activity in warmer areas. Another method that could restore tyrosinase activity is the use of proteasome inhibitors [7]. Furthermore, a study by Halaban et al. demonstrated that co-expression (ectopic expression) of WT protein with temperature-sensitive tyrosinase mutants corrected the mutant conformation defect in an activity-dependent manner [24]. An understanding of the mechanisms by which temperature-sensitive-OCA1B mutants decrease tyrosinase activity would accelerate the search for novel compounds to restore tyrosinase activity.

In conclusion, we have demonstrated, for the first time, that the association of L-DOPA with R422Q and P406L is a is a complex reaction supported by enthalpy and entropy forces similar to that of the WT control. Although we see a change in Tyr activity in R422Q and P406L, we found similar K_m_ values and thermodynamic behavior in WT, R422Q, and P406L. This led us to deduce that the change in activity might be due to the allosteric effect. The analysis of the temperature-dependent kinetics allows us to characterize OCA1B mutants and provides us an easier route to determine the apparent thermodynamic parameters from absorption measurement. This, in turn, enhances our understanding of the mechanisms by which OCA1B mutations decrease Tyr activity, accelerating the search for novel compounds that can recover Tyr activity in OCA1B patients. Finally, in addition to in vitro studies, in vivo studies of similar nature coupled with investigation of other mutants would advance our understanding of mechanisms in OCA1B.

## 4. Materials and Methods

### 4.1. Tyrosinase Expression and Purification

Recombinant truncated human wild-type tyrosinase, WT, and OCA1B-related mutant proteins, R422Q and P406L, were expressed and purified, as previously described [1,2,10,16]. Briefly, the WT (residues 19–469 of the full-length protein), R422Q, and P406L proteins were produced in whole insect *Trichoplusia ni (T. ni)* larvae. The infected larvae, frozen at −80 °C, were homogenized in 5× (*v/w*) lysis buffer (20 mM sodium phosphate, pH 7.4, 500 mM NaCl, 20 mM imidazole, 2 mM MgCl_2_, 0.2 mg/mL lysozyme from chicken egg whites (Sigma-Aldrich, Oakville, ON, Canada), 25 µM 1-phenyl-2-thiourea (Sigma-Aldrich, Saint Louis, MO, USA), 40 µg/mL DNAse (Thermo Fisher Scientific, Waltham, MA, USA), and protease inhibitor tablets (Roche Diagnostics, San Francisco, CA, USA). The homogenates were incubated for 30 min and sonicated for 10 min at room temperature. The lysates were centrifuged at 8000 rpm for 30 min at 4 °C, and then filtered to obtain the supernatants, which were diluted in a 1:1 ratio with affinity binding buffer (20 mM sodium phosphate, pH 7.4, 500 mM NaCl, and 20 mM imidazole). The C-terminal 6-His tagged WT, R422Q, and P406L proteins were purified by IMAC followed by SEC at room temperature using ÄKTAxpress and ÄKTA pure protein purification workstations, respectively (GE Healthcare, Silver Spring, MD, USA).

Soluble extracts were loaded on 5 mL His-Trap FF crude IMAC column (GE Healthcare, Silver Spring, MD, USA) equilibrated with affinity binding buffer and eluted with affinity elution buffer (20 mM sodium phosphate, pH 7.4, 500 mM NaCl, and 500 mM imidazole). Proteins were further purified by SEC using Sephacryl S-300 HR 26/60, Superdex 200 Increase GL 10/300, and Superdex 75 16/60 HR columns (GE Healthcare, Silver Spring, MD, USA). The columns were calibrated using SEC standards (Bio-Rad, Hercules, CA, USA): bovine thyroglobulin, bovine gamma-globulin, chicken ovalbumin, horse myoglobin, and vitamin B12. Fractions containing the WT, R422Q, and P406L proteins were identified using SEC profiles and colorimetric activity test described below, and then concentrated using Amicon Ultra 10,000 MWCO centrifugal filter units (Merc Millipore, Burlington, MA, USA). The protein purity and identity were assessed using SDS-PAGE (Bio-Rad) and Western blot analysis using anti-Tyr (T311) antibodies (Santa Cruz Biotechnology, Dallas, TX, USA), respectively. After each step of purification, the WT, R422Q, and P406L proteins were quantified at A_280nm/260nm_ using a NanoDrop 2000 UV-Vis Spectrophotometer (Thermo Scientific, Waltham, MA, USA).

### 4.2. Tyrosinase Colorimetric Assay

Tyr diphenol oxidase activity was measured spectrophotometrically at 475 nm using a SpectraMax i3 multi-mode microplate reader detection platform (Molecular Devices, San Jose, CA, USA) as previously suggested [25,26]. The reaction mixture containing 0.05 mg/mL Tyr and 3 mM L-DOPA substrate in 10 mM sodium phosphate buffer, pH 7.4, was incubated for 30 min at 37 °C and monitored by measuring dopachrome formation at 475 nm (ε_dopachrome_ = 3700 M^−1^ cm^−1^).

### 4.3. Michaelis–Menten Kinetics

The diphenol oxidase reaction rate (V_max_) was determined using 0.05 mg/mL Tyr and L-DOPA as a substrate at concentrations of 0.094, 0.188, 0.375, 0.75, 1.5, 3, and 6 mM. All assays were performed in duplicates in 10 mM sodium phosphate buffer (pH 7.4) for 1 h at 28, 31, 37, and 43 °C. The reactions were monitored for dopachrome formation at 475 nm with a SpectraMax i3 using a microtiter 96-well plate.

Beer–Lambert law, with ε dopachrome = 3700 M^−1^ and L = 0.3 cm, was used to convert the absorbance measurements (mOD) of dopachrome formation at 475 nm to concentration measurements (mM). The first twenty minutes of every reaction was used to find the initial velocity, V_initial_. Then, the Michaelis–Menten constant, K_m_, and the maximum rate of reaction, V_max_, were calculated from the Michaelis–Menten plots, which were fitted with nonlinear polynomial function on OriginPRO Software (version 7.5, OriginLAB Corporation, Boston, MA, USA). The enzyme turnover (*k_cat_*), which is defined as the number of substrate molecules turned over to product per enzyme per minute, was derived from V_max_/E_t_, where E_t_ is the total amount of enzyme in nmol.

### 4.4. The Van’t Hoff Analysis

The thermodynamic signature of dopachrome production was determined by measurement of the Michaelis–Menten constant (K_m_) at 28, 31, 37, and 43 °C followed by an analysis using the Van’t Hoff equation. The Michaelis–Menten constant K_m_ matches the dissociation constant if it is assumed that the formation of the product from tyrosinase-L-DOPA complex occurs at a much slower rate as compared with the rate of dissociation of tyrosinase-L-DOPA complex (i.e., k_2_ *<<* k_−1_ of the reaction) [17]. In that condition, the thermodynamic parameters were considered apparent values. Therefore, the K_m_ values at the four temperatures were graphed on a Van’t Hoff plot, and then a straight line was fit using OriginPRO Software (version 7.5, OriginLAB Corporation) to analyze the data. The linear fit in the Van’t Hoff plot could be interpreted with the following equation:

ln (K_a_/[E]) = (ΔH/R) 1/T − ΔS/R

where K_a_ is the association constant (K_a_ = [E]/K_m_), K_m_ is the Michaelis–Menten constant, [E] is the Tyr concentration in mM, T is temperature measured in Kelvin (K), R is a gas constant (R = 8.31 J/K/mol), ΔH/R is the slope, and ΔS/R is the y-intercept of the line. Using ΔH, ΔS, and T, the ΔG was calculated for each temperature using the Gibbs free energy equation *(*ΔG = ΔH − TΔS). To obtain ΔΔG values, the ΔG of WT was subtracted from the *ΔG* of mutants. To calculate the free energy changes (ΔΔG_Obs_ − ΔΔG_Calc_) caused by dopachrome association, the ΔΔG from docking simulations (ΔΔG_Cal*c*_) was subtracted from the ΔΔG of Michaelis–Menten kinetics data (ΔΔG_Obs_).

### 4.5. Mutant Modeling, Simulation, and Docking

A human tyrosinase structure (Tyr) was downloaded from the ocular proteomics website (https://neicommons.nei.nih.gov/#/proteome, accessed on 1 June 2021). Global mutagenesis was conducted on Tyr, and each mutant was characterized by a thermodynamic change in Gibbs free energy (∆∆G), which was calculated by the semi-empirical method FoldX and by unfolding mutation screen [27,28]. Tyr is a human homology model of the intra-melanosomal domain of human tyrosinase (residues 19–469) and was refined by 2 ns of molecular dynamics (MD) using md_run.mcr script incorporated to a molecular-graphics, -modeling, and -simulations program Yasara [29,30] (http://www.yasara.org, accessed on 1 June 2021). Two OCA1B mutants, R422Q and P406L, were created using the Edit > Swap > Residue function on the Tyr structure in YASARA. The Tyr, R422Q, and P406L structures were subjected to 100 ns of MD in YASARA. The cell size extended to 10 Å beyond each side of the protein in the shape of a cube with dimensions 92.9 × 92.9 × 92.9 Å. The default pH was set to the value of 7.4, assuming a fixed protonation for all residues. The ion concentration was added as a mass fraction with 0.9% NaCl, and pressure control was adapted to allow for a variable water density to ensure the pressure remained at 1 bar at four different temperatures: 25, 31, 37, and 43 °C. An AMBER14 forcefield was used with a timestep of 2.5 fs. After MD, the different PDB files were subjected to molecular docking using YASARA’s dock_run.mcr script. It was modified to allow for 200 docking runs for each receptor-ligand pair. YASARA employs AutoDock Vina [31], a gradient-optimization method. The tyrosinase protein receptor was held rigid, and the L-DOPA ligand was held flexible. The script uses a statistical scoring function to give each of the receptor-ligand binding conformations binding energy and binding affinity without an assumption about pH or salt conditions. The binding affinities and experimental enzyme concentration were used to determine association constants at four different temperatures and to create a Van’t Hoff plot.

### 4.6. Statistical Analysis

All experiments were performed in duplicates and error bars represent standard deviations from the mean. The K_m_, V_max_, *k_cat_* values and other kinetic parameters were averaged and standard errors were determined in each case. To obtain the errors for the additional kinetic and thermodynamic parameters derived from the experimental data, the proper error propagation formulas were utilized.

## Figures and Tables

**Table 1:** Temperature-dependent dopachrome production in WT, R422Q, and P406L diphenol oxidase reaction.

Protein	Dopachrome Production Rate, P_d_ (mM/°C)	Dopachrome Production at 0 °C, D_o_ (mM)	Adj. R^2^
**WT**	0.009	0.094	0.990
**R422Q**	0.007	0.098	0.998
**P406L**	0.007	0.071	0.978

The parameters shown here are derived from the equation of the linear fit (D = P_d_ × T − D_o_) shown in Supplementary Materials Figure S2. Here D, P_d_, D_o_, and T are dopachrome production, dopachrome production rate, dopachrome production at 0 °C, and temperature (°C), respectively.

**Table 2:** Enthalpy and entropy of WT, R422Q, and P406L obtained from Van’t Hoff analysis of the diphenol oxidase reactions and computational simulations.

	ΔH_Obs_ (kJ/mol)	ΔS_Obs_ (kJ/mol·K)	Adj. R^2^	ΔH_Calc_ (kJ/mol)	ΔS_Calc_ (kJ/mol·K)	Adj. R^2^
WT	−23.15 ± 6.27	−0.12 ± 0.02	0.81	−43.11± 2.26	−0.16 ± 0.01	0.99
WT *	−21.26 ± 6.18	0.06 ± 0.02	0.75	−25.72 ± 13.96	0.08 ± 0.05	0.44
R422Q	−22.48 ± 5.51	−0.11 ± 0.02	0.84	−30.16± 3.37	−0.13 ± 0.01	0.96
P406L	−26.12 ± 0.83	−0.13 ± 0.00	0.99	−33.66 ± 1.91	−0.14 ± 0.01	0.99

Here ΔH_Obs_ and ΔS_Obs_ are the change in enthalpy and entropy observed from the diphenol oxidase reaction, respectively, while ΔH_Calc_ and ΔS_Calc_ are the change of enthalpy and entropy calculated from the docking simulations, respectively. * Previously published data from Table 2 of Young et al. [17] are shown for WT.

## Data Availability

The data presented in this study are openly available in IJMS at this paper.

## Supplementary Materials

The following are available online at https://www.mdpi.com/article/10.3390/ijms22157771/s1.
