# Supplementary material for: Characterization of Temperature-Dependent Kinetics of Oculocutaneous Albinism-Causing Mutants of Tyrosinase
Source: Int J Mol Sci. 2021 Jul 21;22(15):7771. doi: 10.3390/ijms22157771 (PMC8346126; doi:10.3390/ijms22157771)
Supplement: Supplementary file 1 [file ijms-22-07771-s001.zip › ijms-1269101 SI revised.pdf]

Characterization of Temperature-Dependent Kinetics of Oculocutaneous Albinism Causing Mutants of Tyrosinase

Samuel A. Wachamo, Milan H. Patel, Paul K. Varghese, Monika B. Dolinska, and Yuri V. Sergeev

Supplementary Materials

Supplementary Tables

Supplementary Table S1. Kinetic Parameters of WT, R422Q, and P406L catalyzed reactions.

Diphenol Oxidase Activity				
28 °C	K_m mM	V_{max} nmol/min	kcat min-1	kcat/Km mM-1 min-1
WT	0.21 ± 0.04	0.50 ± 0.02	5.55 ± 0.22	26.43 ± 5.14
R422Q	0.19 ± 0.03	0.28 ± 0.01	3.11 ± 0.11	16.37 ± 2.65
P406L	0.20 ± 0.03	0.40 ± 0.02	4.44 ± 0.22	22.20 ± 3.51
31 °C				
WT	0.21 ± 0.04	0.66 ± 0.03	7.33 ± 0.33	34.90 ± 6.83
R422Q	0.18 ± 0.03	0.35 ± 0.01	3.88 ± 0.11	21.56 ± 3.64
P406L	0.22 ± 0.03	0.45 ± 0.02	5.00 ± 0.22	22.73 ± 3.26
37 °C				
WT	0.30 ± 0.06	0.91 ± 0.04	10.11 ± 0.44	33.70 ± 6.90
R422Q	0.25 ± 0.04	0.51 ± 0.02	5.66 ± 0.22	22.64 ± 3.73
P406L	0.27 ± 0.04	0.58 ± 0.02	6.44 ± 0.22	23.85 ± 3.63
43 °C				
WT	0.30 ± 0.06	1.12 ± 0.06	12.44 ± 0.66	41.47 ± 8.58
R422Q	0.27 ± 0.04	0.67 ± 0.03	7.44 ± 0.33	27.56 ± 4.26
P406L	0.33 ± 0.04	0.79 ± 0.03	8.78 ± 0.33	26.61 ± 3.38

Here K_m, the Michaelis-Menten constant measures the affinity of the substrate for the enzyme; V_{max}, the maximum rate at which a substrate will be converted to the product once bound to the enzyme; k_{cat}, the enzyme turnover defines the number of substrate molecules turned over per enzyme molecule per minute. The enzyme turnover is equal to V_{max}/E_t, where E_t is the concentration of enzyme in nmol; k_{cat}/K_m, enzyme efficiency.

Supplementary Table S2. The dissociation constant (K_d) values obtained from docking simulations. Here the K_d values for 25, 31, 37, and 43 °C are shown for WT, R422Q, and P406L.

K_d (mM)	25 °C	31 °C	37 °C	43 °C
WT	0.018	0.027	0.036	0.048
R422Q	0.037	0.047	0.055	0.076
P406L	0.030	0.038	0.053	0.064

Supplementary Table S3. Temperature-dependent free energy changes (ΔΔG) caused by dopachrome associations. To calculate the free energy changes, the ΔΔG from docking simulations ($\Delta\Delta G_{Calc}$) was subtracted from the ΔΔG of Michaelis-Menten kinetics ($\Delta\Delta G_{Obs}$). Here ΔΔG stands for the free energy change obtained by subtracting the ΔG of the WT from the ΔG of mutant variants at different temperatures.

	$\Delta\Delta G_{Obs}$ – ΔΔGCalc					
	28 °C	31 °C	37 °C	43 °C	Average	Standard Deviation
R422Q	-2.011	-1.945	-1.777	-1.610	-1.836	0.180
P406L	-1.032	-0.942	-0.762	-0.583	-0.830	0.199

Supplementary Table S4. Linear fit of temperature-dependent free energy changes (ΔΔG) caused by dopachrome associations. Free energy change values (ΔΔG) shown in Supplementary Table S3 were graphed and a straight line was fit to obtain the parameters shown below. The
ΔΔG value increases linearly with temperature according to the following equation $E = E_d{*}T - E_o$ (Figure 7). Here E, E_d, E_o, and T denote the change in ΔΔG ($\Delta\Delta G_{Obs}$ - $\Delta\Delta G_{Calc}$), rate of change in ΔΔG, change in ΔΔG at 0 ºC, and temperature (28, 31, 37, or 43 ºC), respectively.

	Rate of Change in ΔΔG (kJ/mol/°C), E_d	Change in ΔΔG at 0 °C (kJ/mol), E_o	Adj. R^2
R422Q	0.027	2.775	0.999
P406L	0.030	1.870	1.000

Supplementary Figures

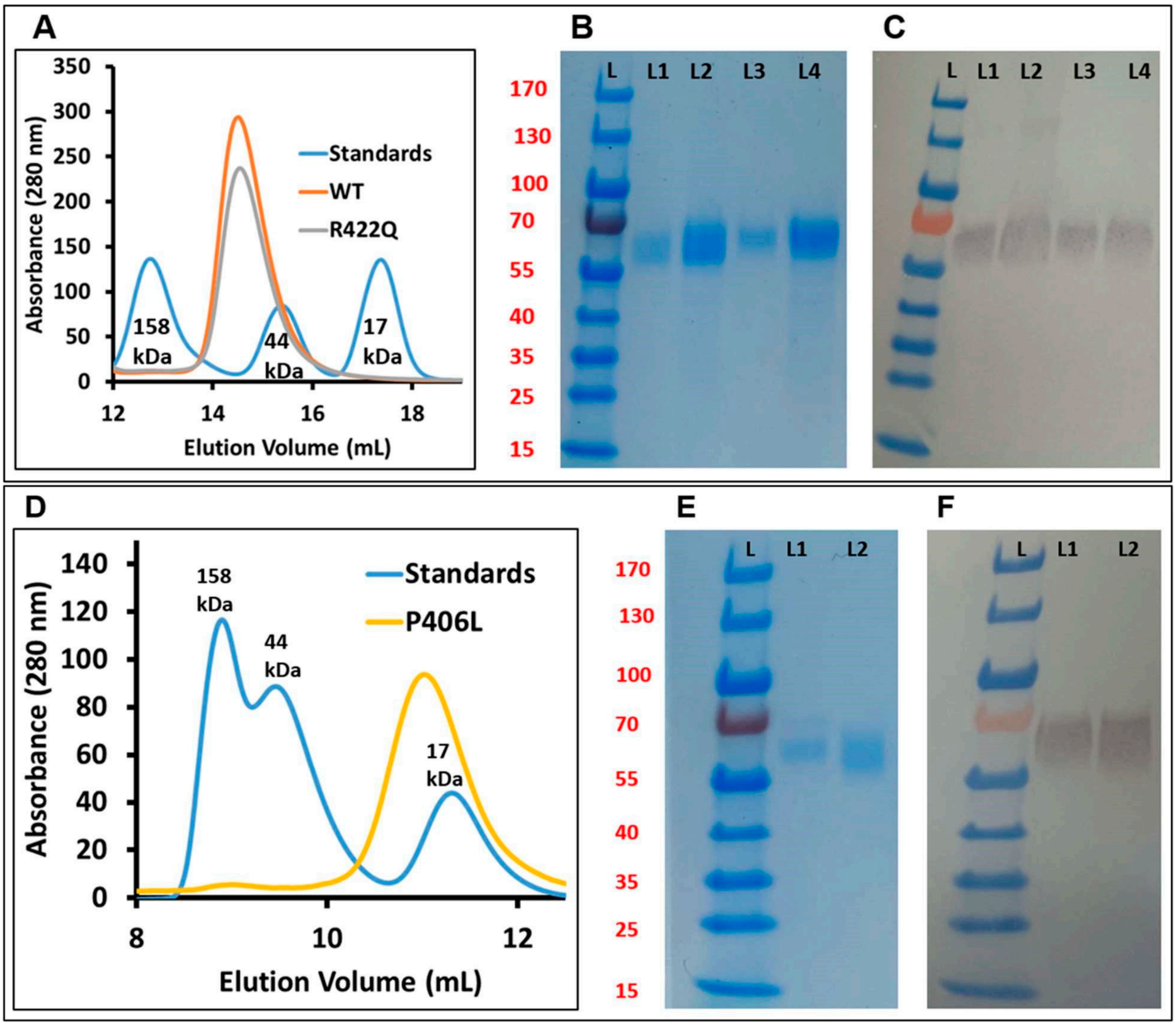

Supplementary Figure S1. Purification and identification of recombinant WT, and OCA1B related mutant proteins (R422Q and P406L). Panels A and D show the purification using Superdex 200 Increase GL 10/300 and Superdex 75 16/60 HR columns, respectively, in the

ÄKTA pure workstation. The blue peaks correspond to the standard proteins Gamma-globulin (158 kDa), ovalbumin (44 kDa), and myoglobin (17 kDa) while the orange, gray and yellow peaks correspond to WT, R422Q, and P406L, respectively. Panels B and C represent the SDSPAGE and Western Blot of WT and R422Q. Lanes L, L1, L2, L3 and L4 correspond to the ladder, 0.05 mg/mL WT, 0.2 mg/mL WT, 0.05 mg/mL R422Q, and 0.2 mg/mL R422Q, respectively. Panels E and F feature the SDS-PAGE and Western Bolt of P406L. Lanes L, L1, and L2 correspond to the ladder, 0.05 mg/mL P406L, and 0.05 mg/mL WT, respectively. The SDS-PAGE and Western Blot were performed using proteins obtained after the third step of purification using Superdex 200 Increase GL 10/300 column in all cases. The left lanes of Panels

B, C, E, and F show the ladder with molecular weights shown in kDa.

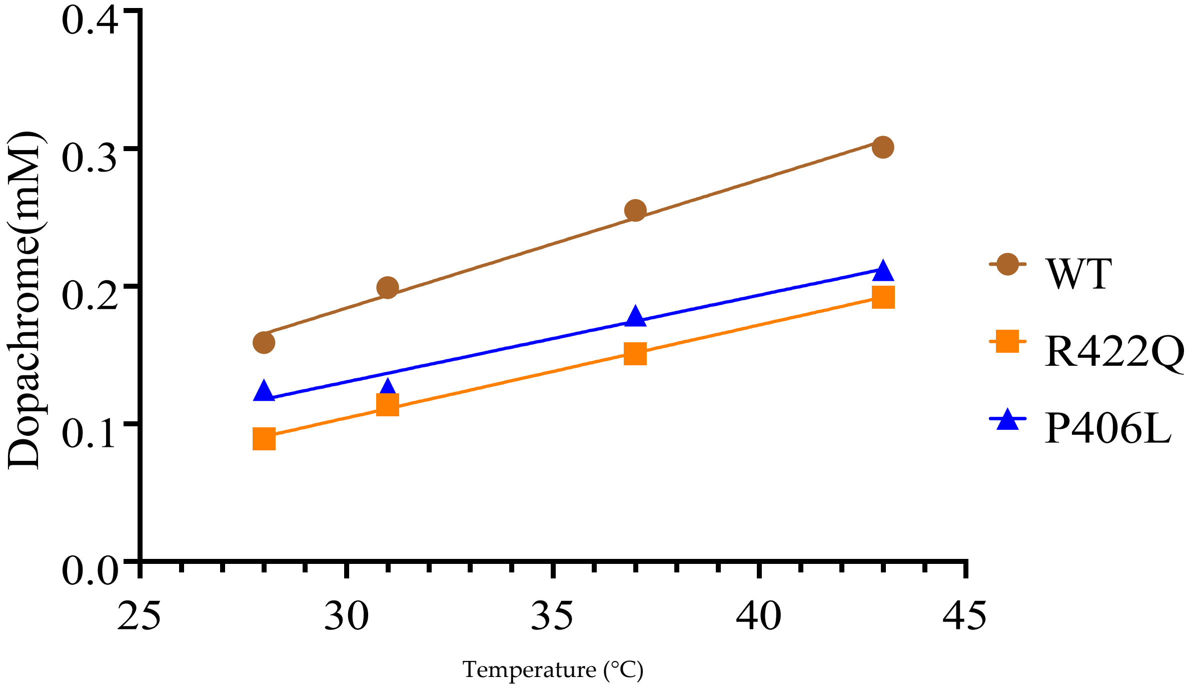

Supplementary Figure S2: The role of temperature in the Tyr reaction and production of dopachrome. WT, R422Q, and P406L are shown in brown, orange, and blue, respectively. The diphenol oxidase activities were determined by using 0.05 mg/mL Tyr and 3 mM L-DOPA as a substrate. The absorbance measurements (mOD) of dopachrome production at 475 nm were obtained after running the reactions at the temperature conditions for 30 minutes. The production of dopachrome increases linearly with temperature according to the following equation ($D = P_d*T - D_o$). Here D, P_d, D_o, and T are dopachrome production, dopachrome production rate, dopachrome production at 0 °C, and temperature (ºC), respectively. The brown ($D = 0.009*T – 0.094$, $R^2 = 0.990$), orange ($D = 0.007*T – 0.098$, $R^2 = 0.998$) and blue ($D = 0.007*T – 0.071$, $R^2 = 0.978$) lines are for WT, R422Q and P406L, respectively. All experiments were performed in duplicates and error bars represent standard deviations.

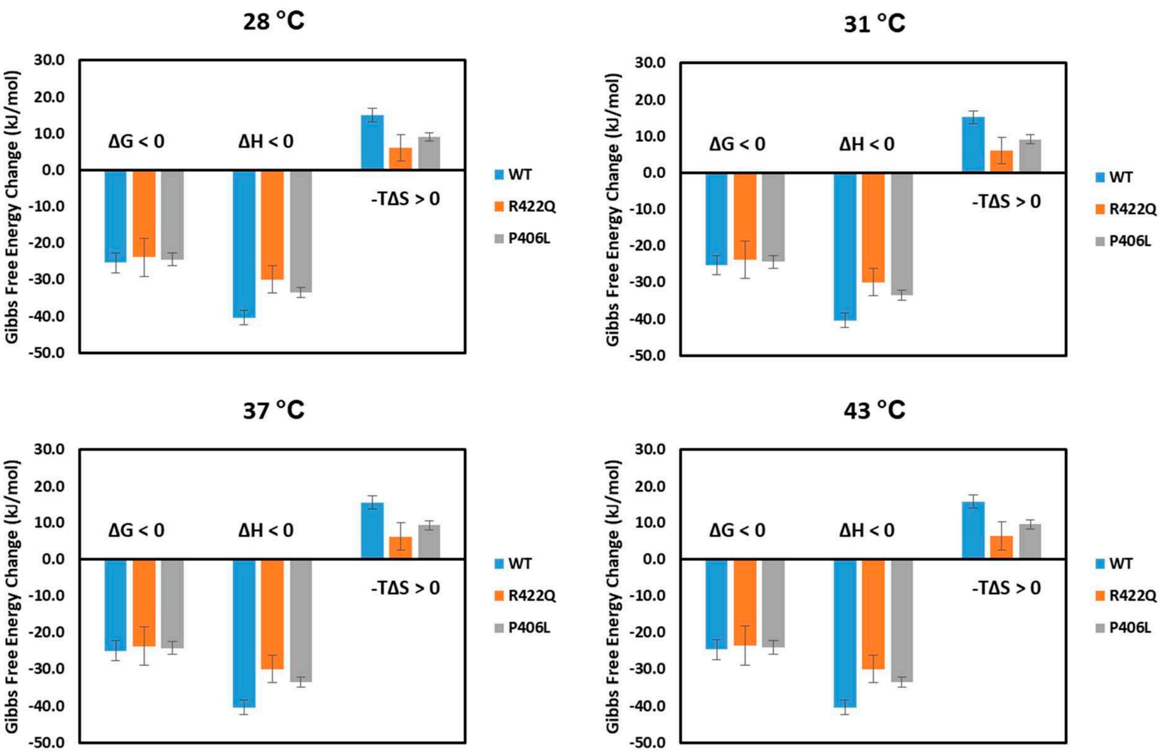

Supplementary Figure S3: The thermodynamic signatures obtained from computational simulations of the association of L-DOPA with WT (blue), R422Q (orange), and P406L (gray) suggest complex reactions controlled by enthalpy and entropy. Error bars represent errors propagated from linear regressions of van't Hoff plots.

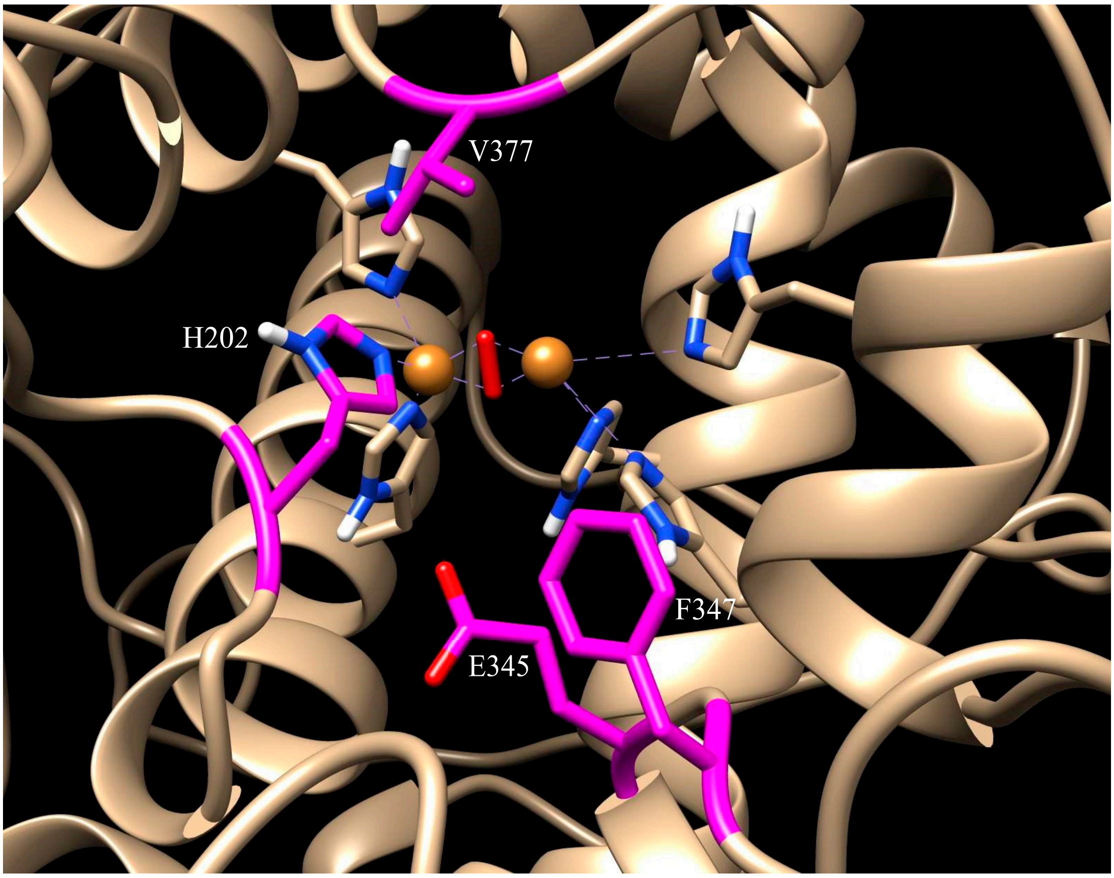

Supplementary Figure S4: The residues of Tyr involved in substrate docking for WT, R422Q, and P406L at all four temperatures are highlighted in magenta on the initial homology model of Tyr. These include H202, E345, F347, and V377.

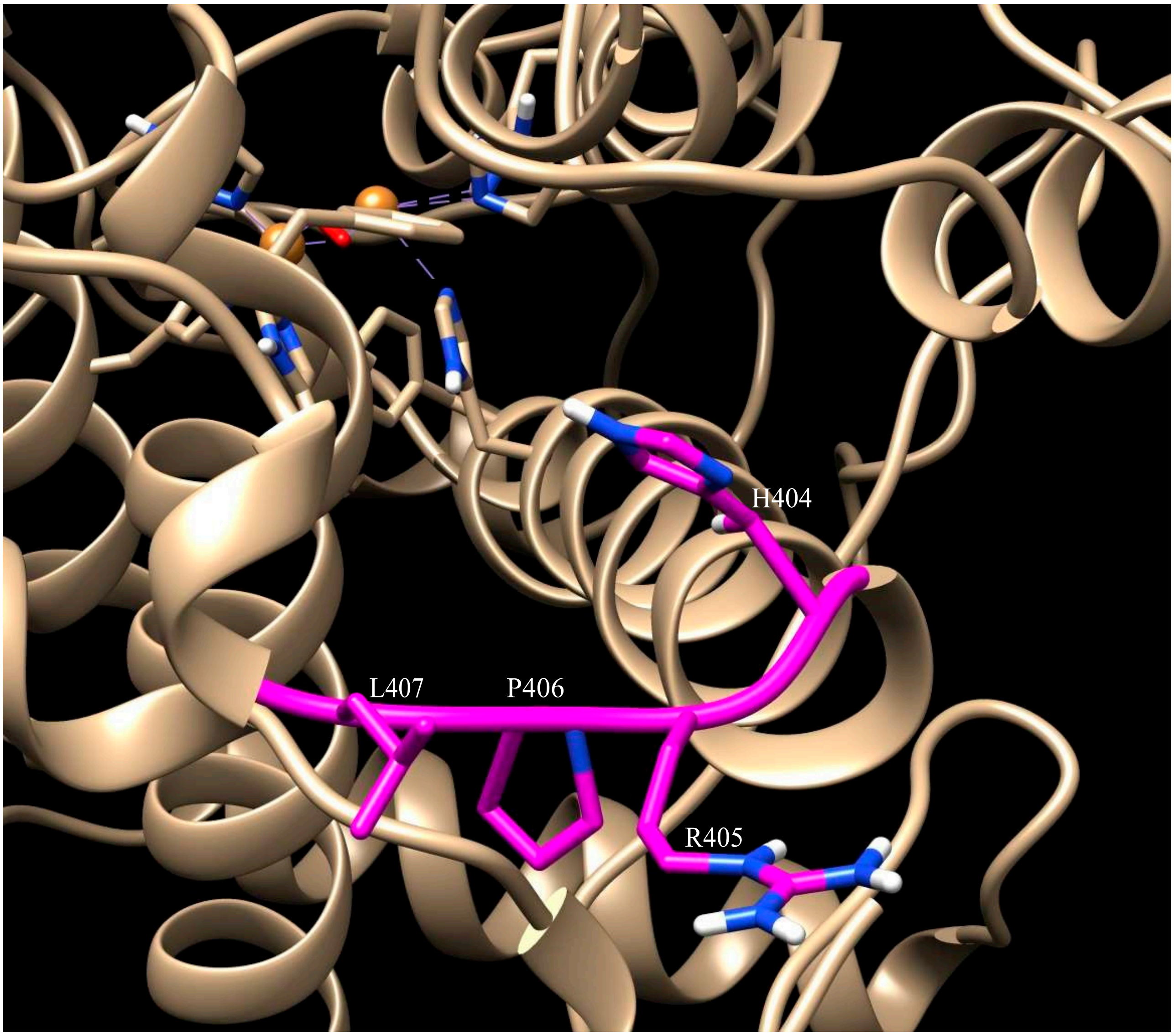

Supplementary Figure S5: The P406 environment shown in the initial homology model. P406 is located in a four-residue long loop located in between two alpha-helices. The mutation from proline to leucine could allow the preceding helix to extend until the end of the succeeding helix. The preceding helix contains copper-coordinating histidines, and an extension of this helix could result in a non-native structure of Tyr, thereby leading to active site structural alteration.

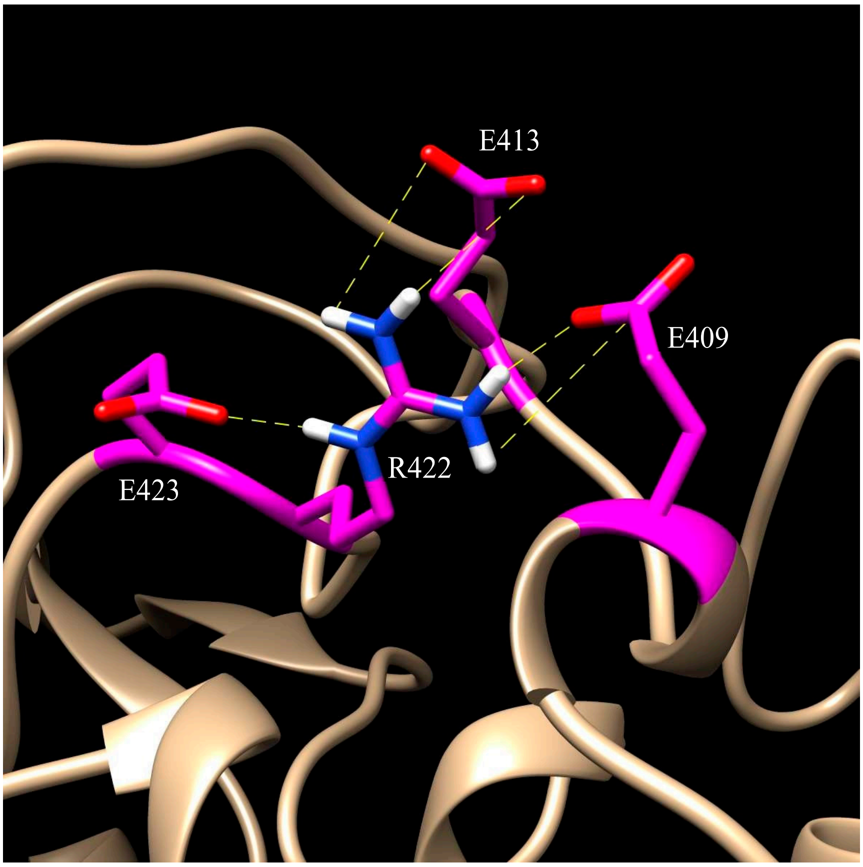

Supplementary Figure S6: The R422 environment shown in the initial homology model. R422 is located within a glutamate pocket (E409, E413, E423), allowing for the formation of up to five hydrogen bonds. The mutation from arginine to glutamine limits hydrogen bond formation to two total bonds, decreasing the thermodynamic stability of the region.

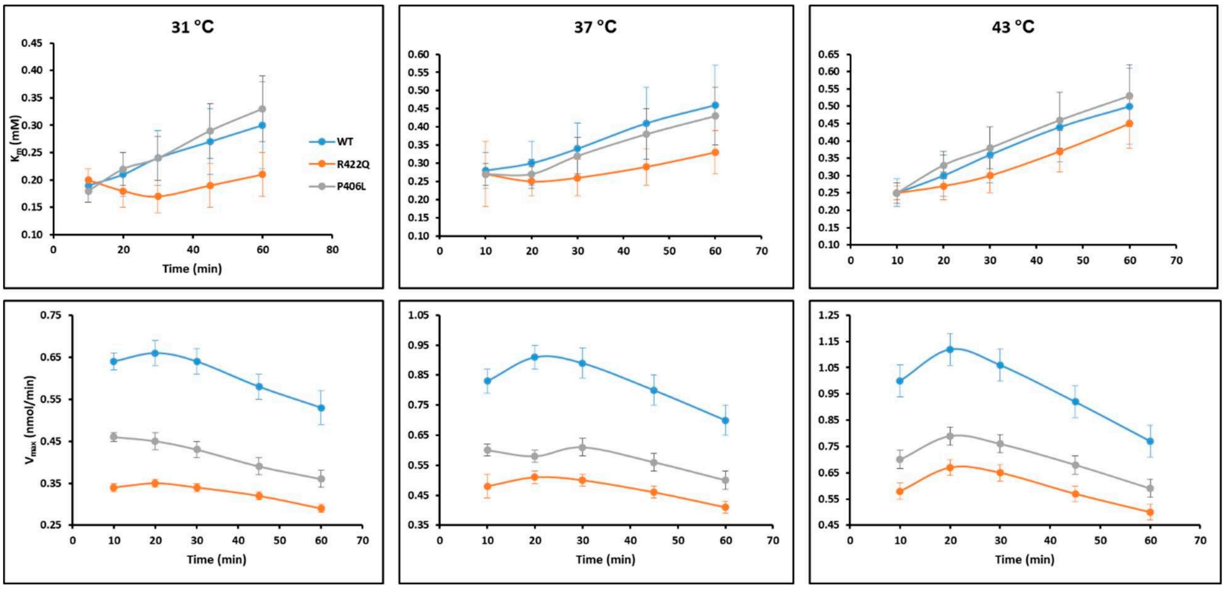

Supplementary Figure S7: Temperature-dependent changes of kinetic parameters (K_m and V_{max}). The K_m (top panels) and V_{max} (bottom panels) values of WT, R422Q, and P406L were determined over an hour of diphenol oxidase reaction. The Michaelis-Menten plots of diphenol oxidase activity were obtained as a function of L-DOPA concentration at 31, 37, and 43 °C. The diphenol oxidase reaction was run for an hour, and then the Michaelis-Menten plots were graphed for 10, 20, 30, 45, and 60 minutes of the reaction. Each data point represents the average of duplicate measurements, with error bars representing standard deviations.

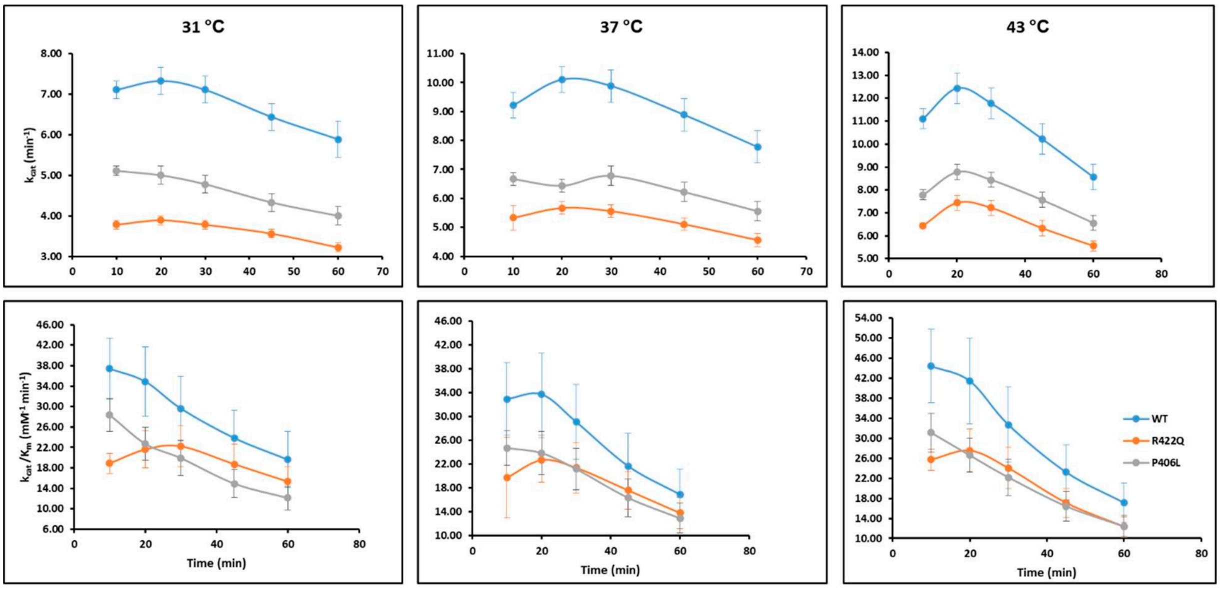

Supplementary Figure S8: Temperature-dependent changes of kinetic parameters (turnover number and catalytic efficiency). The turnover number (top panels) and catalytic efficiency (bottom panels) values of WT, R422Q, and P406L were determined over an hour of diphenol oxidase reaction. The Michaelis-Menten plots of diphenol oxidase activity were obtained as a function of L-DOPA concentration at 31, 37, and 43 °C. The diphenol oxidase reaction was

run for an hour, and then the Michaelis-Menten plots were graphed for 10, 20, 30, 45, and 60 minutes of the reaction. Each data point represents the average of duplicate measurements, with error bars representing standard deviations.

WT

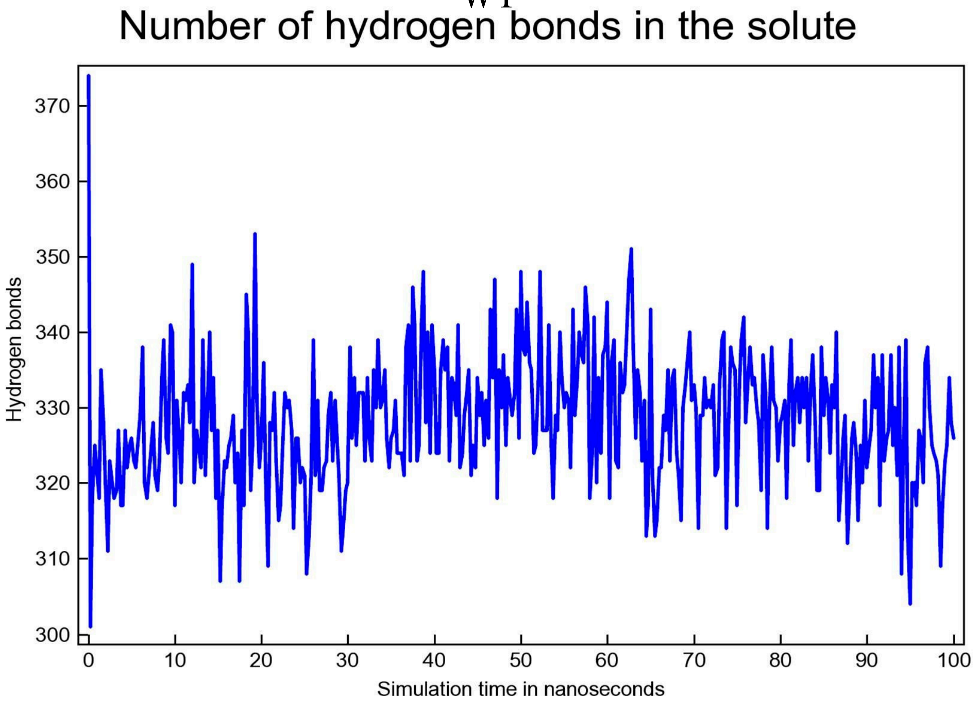

R422Q

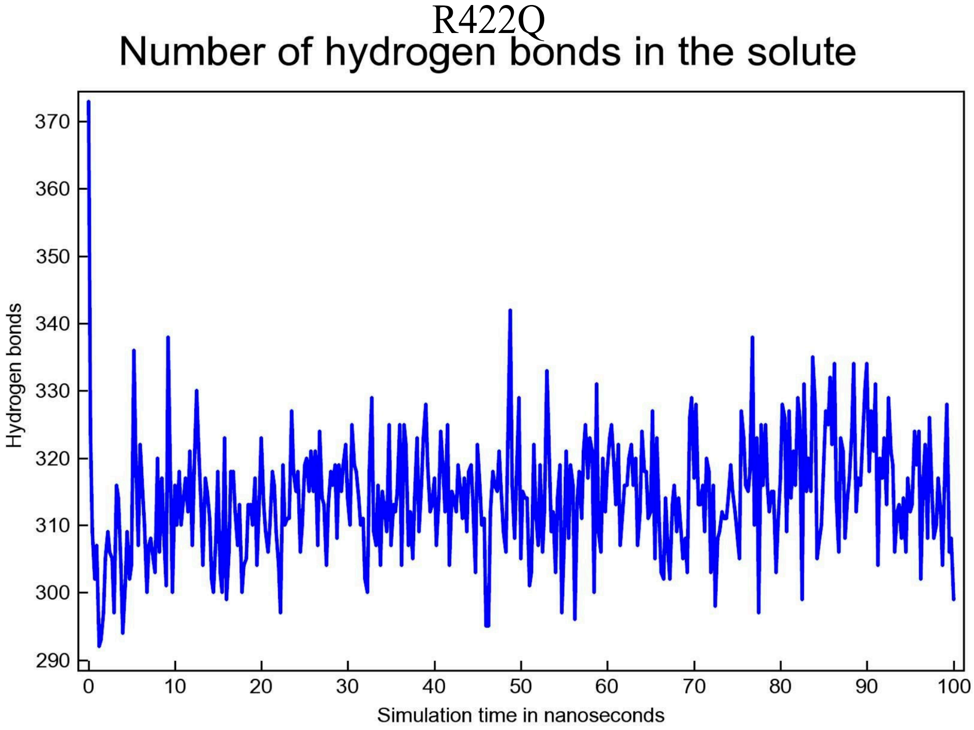

Supplementary Figure S9: Graphs of the total number of hydrogen bonds in the protein structure for WT Tyr and R422Q. R422Q is expected to lose transient hydrogen bonds with surrounding residues including E409, E413, and E423. On average, R422Q has fewer hydrogen bonds than WT Tyr.

A WT

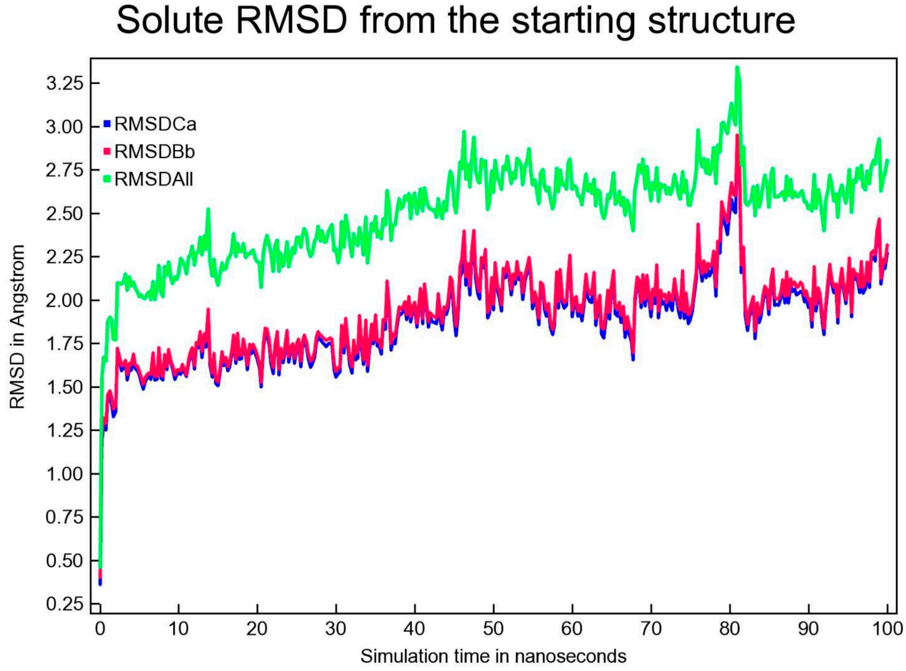
Solute RMSD from the starting structure
RMSDCa
RMSDBb
RMSDAll
RMSD in Angstrom
Simulation time in nanoseconds

B P406L

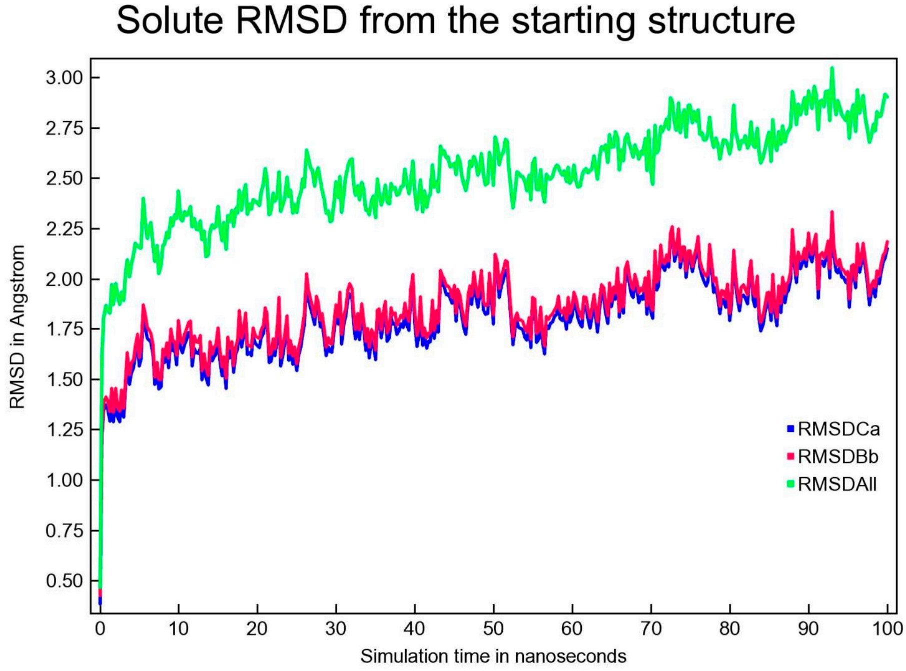
Solute RMSD from the starting structure
RMSDCa
RMSDBb
RMSDAll
RMSD in Angstrom
Simulation time in nanoseconds

C

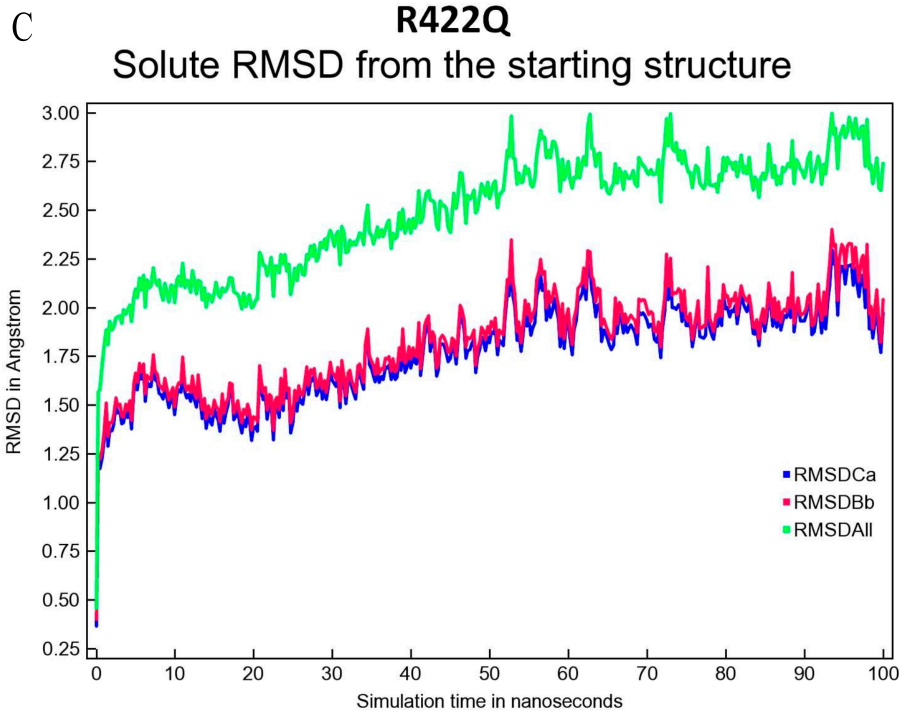

Supplementary Figure S10: RMSD of WT (A), P406L (B), and R422Q (C) throughout the simulation. RMSD graphs indicate that the simulations for all three structures are stable.

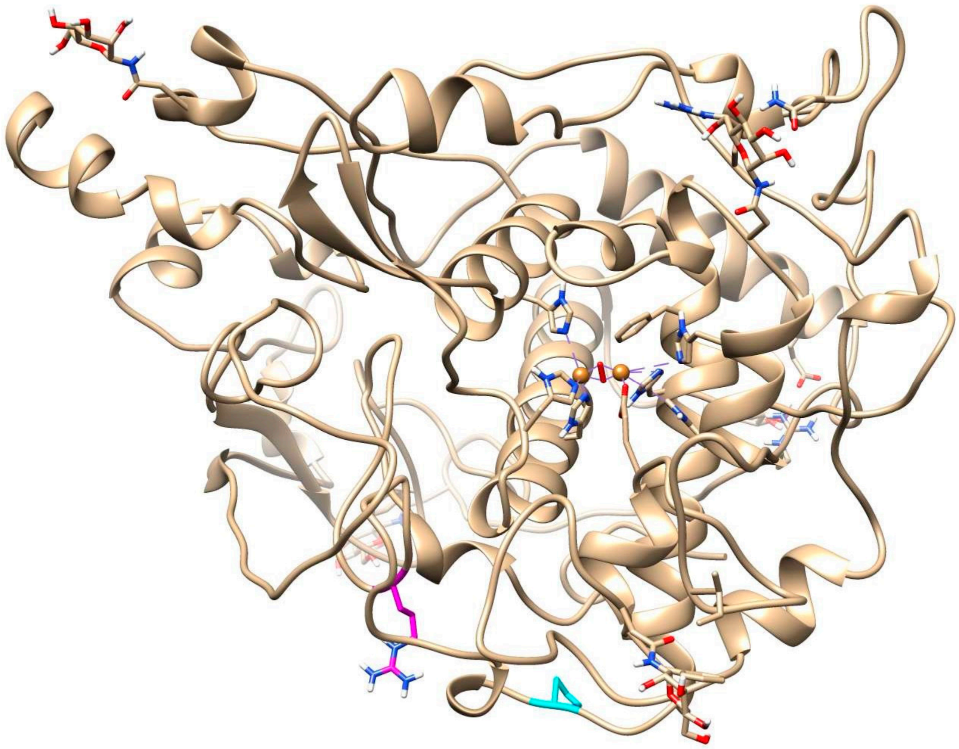

Supplementary Figure S11. The overall structure of glycosylated Tyr homology model is shown with the active site facing forwards. P406 is highlighted in cyan and R422 is highlighted in magenta. Five glycans are included at the various Asn sites.
